# Low math anxiety prevents math achievement decline for urban but not rural Chinese schoolchildren

**DOI:** 10.3389/fpsyg.2025.1665098

**Published:** 2026-01-13

**Authors:** Zhen Ying, He Chungang, Yu Haiying, Anna Pavlova, Artem Malykh, Sergey Malykh

**Affiliations:** 1 Mudanjiang Normal University, Mudanjiang, China; 2 Russian Academy of Education, Moscow, Russia; 3 Ural Institute of Humanities, Ural Federal University, Ekaterinburg, Russia

**Keywords:** achievement gap, maternal education, math achievements, math anxiety, nonverbal intelligence, rural vs. urban education

## Abstract

Across a number of countries, rural schoolchildren systematically underperform in math in comparison with their urban counterparts. To plan targeted interventions, it is important to understand if there are any differences in factors influencing math performance in rural and urban areas. The purpose of the study was to compare the association between math performance, socio-demographic variables, nonverbal intelligence, and math anxiety in rural and urban China. The sample consists of 1,412 urban schoolchildren (51% females, M = 12.04) and 1,032 rural schoolchildren (50% females, M = 13.63). Raven’s Progressive Matrices were used to measure nonverbal intelligence, and the Abbreviated Math Anxiety Scale served as a measurement tool for math anxiety. Overall, nonverbal intelligence is positively associated with math performance, whereas math anxiety is negatively associated. In contrast to the subtle decline in math performance with age in urban regions, in rural areas, math performance deteriorates sharply, widening the achievement gap over time. Notably, high nonverbal intelligence, low math anxiety, and high maternal education hinder the decline in math performance in urban areas, whereas in rural areas, only high nonverbal intelligence attenuates the decline. Furthermore, in urban areas, females show lower math achievement and higher math anxiety than males, while no gender differences are observed for rural areas. Potential explanations of the difference between rural and urban contexts and practical implementation are discussed.

## 1 Introduction

Math achievements in school are a strong predictor of later academic outcomes (Reddy et al., 2012), career aspirations (Shapka et al., 2006), choice of Science, Technology, Engineering, and Mathematics (STEM) professions (Wang, 2013), retention in STEM education (Leuwerke et al., 2004), and socio-economic status in adulthood (Ritchie and Bates, 2013). Yet, depending on population and diagnostic criteria, from 4 to 15% of schoolchildren consistently experience math difficulties (Wang et al., 2024; Jabeen et al., 2021; Morsanyi et al., 2018; Lewis et al., 1994). Moreover, math achievements are shown to decline with age (Gottfried et al., 2007) and be particularly low in girls (Ellison and Swanson, 2010; Else-Quest et al., 2010; Cimpian et al., 2016; Reilly et al., 2015), immigrants (Huang, 2000; Martin et al., 2012), children from poor socio-economic backgrounds (Hopkins, 2005; Short and McLean, 2023; Baird, 2012; Muskens et al., 2024; Jordan and Levine, 2009), and rural living areas (Ababneh and Kodippili, 2020; Butt and Dogar, 2014; Tayyaba, 2012; Betancur et al., 2023). In particular, the math achievement gap between rural and urban regions was observed in 14 countries (Williams, 2005), and it has widened over time (Graham and Provost, 2012). Among possible reasons for this gap are low value placed on math and academic achievement in rural areas (Reeves, 2012), as well as lack of economic (Williams, 2005) and environmental (Betancur et al., 2023; Irvin et al., 2017; Graham and Provost, 2012) resources.

A variety of cognitive, socio-emotional, and socio-demographic factors explain individual and group differences in math achievements (see Myers et al., 2017; Moustafa et al., 2017; Robson et al., 2020; Jordan and Levine, 2009, for review and meta-analyses).

Math anxiety, defined as a tendency to feel tension and fear while solving math tasks, is one of the key socio-emotional traits explaining variance in math achievements. Several studies pointed out the negative association between math anxiety and math achievements across different ages, genders, and cultural contexts (see Ma, 1999; Zhang et al., 2019; Barroso et al., 2021; Caviola et al., 2021 for meta-analysis; Chang and Beilock, 2016; Dowker et al., 2016; Foley et al., 2017; Ramirez et al., 2018; Lau et al., 2024 for a review). Two prominent theories explain the nature of the relationship between math performance and math anxiety: the “Reduced Competence Theory” developed by Ramirez et al. (2018) suggests that math anxiety is triggered by initial low math ability, while the Processing Efficiency Theory developed by Eysenck and Calvo (1992) claims that math anxiety hinders math performance by overloading cognitive resources, in particular working memory (Lau et al., 2024; Dowker et al., 2016; Foley et al., 2017). As both theories are supported by empirical evidence, a bidirectional association between math achievements and math anxiety was proposed (Carey et al., 2016). Recent longitudinal studies provide empirical support for the bidirectional association between math achievement and math anxiety, implying that both mechanisms work simultaneously, causing a vicious circle (Szczygieł et al., 2024; Aldrup et al., 2019; Gunderson et al., 2017).

The link between math performance and math anxiety is related to both individual and socio-cultural factors (Chang and Beilock, 2016; Luttenberger et al., 2018). Numerous researchers are dedicated to revealing the factors that moderate the relationship between math achievement and math anxiety, yet have yielded controversial results. Thus, the link between math anxiety and math achievements was shown to be stronger for Asian students in comparison with their European peers (Zhang et al., 2019). Conversely, another study (Lee, 2009) reveals that in some Asian countries students demonstrate high math achievements despite high math anxiety, whereas in European countries high math achievements are linked to low math anxiety. Interestingly, one study (Kalaycioğlu, 2015) reveals an association between math anxiety and math achievements in Turkey, Greece, and the USA, but not in Hong Kong, England, or the Netherlands. Some studies show that the association between math anxiety and math achievement is especially pronounced in children with high working memory (Ramirez et al., 2013, 2016), whereas others demonstrate that children with high math anxiety and low working memory are particularly vulnerable to poor performance in math (Soltanlou et al., 2019; Doz et al., 2024). Although there is a lack of studies on math anxiety differences in rural and urban populations, we assume that rural children would experience higher levels of math anxiety, as it was found to be associated with poor math performance and lower value of math (Wang et al., 2021), in particular for Chinese schoolchildren (Long and Li, 2024; Fu, 2025).

Among cognitive factors, general fluid intelligence is strongly associated with math performance (Semeraro et al., 2023; Romero et al., 2024; Blanch, 2015; McGrew and Wendling, 2010; Peng et al., 2019; Primi et al., 2010; Ren et al., 2015; Cormier et al., 2017; Green et al., 2017; Peng et al., 2019), presumably because math requires more abstract thinking than other school subjects (Romero et al., 2024), and due to the high role of fluid intelligence in the development of basic arithmetic skills, number line estimation (Hornung et al., 2014) and the ability to consider multiple relations between different variables (Miller Singley and Bunge, 2014). Moreover, recent genetic and neuroimaging findings show shared neurobiological underpinnings of fluid intelligence and mathematical performance (for review, see Shuang and Mengmeng, 2022). In particular, nonverbal intelligence predicts math achievement in primary (Tikhomirova et al., 2017), secondary (Bouchefra et al., 2022), and high (Tikhomirova et al., 2016) school. The link is especially strong for achievements on high-stakes exams (Tikhomirova et al., 2016) and complex tasks (Peng et al., 2019), as well as for older children and children from families with high SES (Peng et al., 2019). Furthermore, nonverbal intelligence has been found to predict children’s math performance through the mediation of number sense and basic arithmetic ability (Zhou et al., 2022).

Regarding socio-demographic factors, parental, especially maternal education is strongly linked with children’s math performance (Bellon et al., 2022) due to both genetic (Hart et al., 2009) and environmental factors, such as learning materials, stimulation, parental responsiveness (Zadeh et al., 2010), parenting style (Macmull and Ashkenazi, 2019), parental math anxiety (Maloney et al., 2015), and early exposure to an enriched environment at home (Slusser et al., 2018; Zadeh et al., 2010; Blums et al., 2016). The effect of maternal education was also observed in the Chinese cultural context. Specifically, high school students whose mothers hold a graduate degree demonstrate significantly higher math scores than those whose mothers have no formal education (Fan, 2024).

Gender is also linked with math performance, as boys typically outperform girls in math (Lu et al., 2023; Hyde and Mertz, 2009). Recent Chinese studies demonstrate that boys show notably better learning attitudes toward math than girls (Yang et al., 2024) and have higher math achievements (Wang et al., 2022). Due to the prime role of sociocultural factors, such as gender equality and social expectations (Hyde and Mertz, 2009), the magnitude of the link varies across different cultural contexts (Lu et al., 2023). Based on that evidence, we expect that gender differences will be more pronounced in rural contexts, as they are typically associated with more traditional values and roles (Dunstan et al., 2021).

Age is another socio-demographic factor that is strongly linked to math achievements, as math achievements tend to decline as children grow up, in particular during the transition from primary to secondary school. Older students value mathematics less, have a less positive self-concept in mathematics, use learning strategies less frequently, and achieve lower mathematics performance compared to younger students (Rončević Zubković et al., 2021). Moreover, age moderates the association with gender and math, as the gender gap in math achievements and math anxiety widens over time (Rončević Zubković et al., 2021).

Overall, studies suggest an intricate interplay between math achievements, math anxiety, cognitive abilities, and socio-demographic factors. However, few papers are focused on cultural differences in that complex association. In particular, the specificity of that interplay for rural and urban living areas remains largely unexplored. The present study aims to investigate the association between math performance, nonverbal intelligence, math anxiety, maternal education, gender, and age separately for urban and rural areas of China. In particular, four objectives guided the study:

1) To compare math achievements, math anxiety, and nonverbal intelligence prevalence in rural and urban areas within age groups.
2) To investigate gender differences in math achievements, math anxiety, and nonverbal intelligence prevalence in rural and urban areas.
3) To investigate the difference in the link between math anxiety, nonverbal intelligence, maternal education, and math achievements between rural and urban areas.
4) To investigate the difference in the interplay among correlates of math achievements in rural and urban areas.

In line with these objectives, the following hypotheses were formulated:

1) There is a gap in math achievements between rural and urban areas, and the gap is higher for the older age group. Rural schoolchildren show higher math anxiety than their urban counterparts. There are no differences in nonverbal intelligence between areas.
2) In rural areas, gender differences in math achievements, math anxiety, and nonverbal intelligence are more pronounced than in urban areas.
3) In both rural and urban areas, math achievements are positively associated with nonverbal intelligence and maternal education and negatively associated with math anxiety. Differences in the magnitude of the links may be observed.
4) Nonverbal intelligence, math anxiety, and maternal education moderate the association with math achievements and age in both rural and urban areas. Differences in the magnitude of the moderation effect may be observed.

## 2 Materials and methods

### 2.1 Participants

A total sample comprised 2,444 Chinese schoolchildren. Of these, 1,412 (58%) were urban students (51% females, M = 12.04, sd = 1.07), and 1,032 (42%) were rural students (50% females, M = 13.63, sd = 1.16). Among urban students, 136 (10%) are in 4th grade, 564 (40%) are in 5th grade, 486 (34%) are in 6th grade, 167 (12%) are in 7th grade, and 59 (4%) are in 8th grade. Among rural students, 133 (13%) are in 5th grade, 74 (7%) are in 6th grade, 301 (29%) are in 7th grade, and 524 (51%) are in 8th grade. The school entry age is 6 years across all regions, for both urban and rural areas.

### 2.2 Procedure

The samples for this study were drawn from Mudanjiang City, Heilongjiang Province. The city is located in the southeastern part of Northeast China. Geographically, Mudanjiang City is dominated by mountains and hills. The urban area is the Mudanjiang River Valley basin, and the rural area is scattered around Zhangguangcai Ridge and Laoye Ridge. This spatial distribution between urban and rural areas provides a representative geographical context for studying urban–rural educational disparities.

The sample included both urban and rural schools within Mudanjiang City. Urban schools were located in central districts of Mudanjiang (e.g., Aimin and Dong’an) and represented public compulsory education institutions. The students of such schools are mainly children of urban families with household registration whose parents have relatively high educational levels, family cultural capital, and high levels of educational investment.

Rural schools were selected from public compulsory education institutions in nearby counties and towns, such as Ning’an and Hailin. The students of such schools are mostly children of families with agricultural household registration, and the parents’ educational level is generally not high. Some schools included “left-behind children,” whose parents work elsewhere, leaving them under the care of relatives or other family members.

A total of 16 schools (3 urban junior high, five urban primary, three rural junior high, five rural primary) participated in the study. The schools were selected based on the school availability for the research and data collection. Data were collected via online testing sessions conducted in school classrooms. Written informed consent was obtained from participants’ legal guardians or next of kin. The study was carried out in accordance with the Declaration of Helsinki and approved by the Ethics Committee of Mudanjiang Normal University. The survey school student participation rate was 100%.

### 2.3 Instruments

Math achievement scores were obtained from the school administration. Math achievements reflect the score on a final math exam with unified and standardized content for all China districts. The maximum possible score is 100 for elementary school (grades 1 to 6) and 150 for secondary school (grades 7 to 9). To standardize the scale, raw scores were converted to percentages of the maximum possible score.

Students reported their mothers’ education level based on their own understanding. For the analysis requiring maternal education (linear regression with interactions), the missing values (an option “Other/Not clear”) were removed from the sample.

Raven’s Progressive Matrices (RPM, Raven et al., 1998) was used as a measure of nonverbal intelligence. RPM is widely used as a measure of fluid intelligence in the Chinese cultural context (Lynn, 1991; Qiu et al., 2020). RPM consists of 6 blocks of tasks (A, B, C, D, E, F) with 12 items in each block. The difficulty of tasks increases within each block (e.g., A12 is more difficult than A1) and between blocks (e.g., F1 is more difficult than A1). Each task represents a matrix of geometric patterns with a missing element that has to be chosen from multiple options (Raven, 2000). Each response was scored as either correct (1) or incorrect (0). The maximal possible score is 72. RPM demonstrates high construct validity in several studies [correlations with other cognitive measures range from 0.30 to 0.70 (see Burke, 1958, for a review)], as well as high reliability values [Cronbach *α* 0.88–0.93 (Abdel-Khalek, 2005)].

The Abbreviated Math Anxiety Scale (AMAS, Hopko et al., 2003) was used as a measure of math anxiety level. The AMAS was validated for use in the Chinese cultural context (Hongxia et al., 2022). AMAS consists of 9 items. Five of them relate to learning math anxiety and describe situations that emerge during regular math learning, such as “I feel anxious watching the teacher work on an algebraic equation on the blackboard.” Four of them relate to math evaluation anxiety and describe situations of assessment of math abilities, such as “I feel anxious taking an examination on a math score. Participants rated their agreement with each statement on a five-point Likert scale (1 = “strongly disagree” to 5 = “strongly agree”) (Marakshina et al., 2023, 2024). The maximal possible score is 45. The total AMAS scale, as well as the learning math anxiety and math evaluation anxiety subscales, shows high reliability in the Chinese cultural context [Cronbach α 0.66–0.81 (Linna et al., 2024)].

### 2.4 Statistical analysis

Statistical analysis was performed in Python (v 3.9) (Van Rossum and Drake, 2009) and R software (v 4.5.1). The t-test was used to investigate the differences in math achievement, math anxiety, and nonverbal intelligence in urban and rural areas, as well as gender differences in rural and urban areas. We compared rural and urban schoolchildren within particular age groups, that allows to account for difference in age structure between rural and urban subsamples.

Linear regression with interactions among variables was applied separately to the rural and urban subsets to investigate the associations between math achievement, math anxiety, nonverbal intelligence, and maternal education, as well as the interactions between age and math anxiety, nonverbal intelligence, and maternal education. The groups for nonverbal intelligence and math anxiety were formed based on quantiles. For nonverbal intelligence: below or equal to 37—low, 38 to 47—medium, 48 or higher—high. For math anxiety: below or equal to 13—low, 14 to 18—medium, 19 or higher—high. Maternal education starting from a bachelor’s degree was considered high. For linear regression analysis only, age was centered around the mean to enhance the interpretability of the model intercept and the coefficients. The most disadvantaged subgroup (schoolchildren with high math anxiety, low nonverbal intelligence, and low maternal education) was chosen as a baseline group for linear regression analysis. Linear regression analysis was performed in the lavaan R package.

Given the high susceptibility of linear regression models with interactions between variables to statistical artifacts, additional correlational analysis was performed to triangulate the results obtained in linear regression analysis. The Pearson correlation coefficient was used for that purpose.

## 3 Results

Table 1 summarizes the socio-demographic description of rural and urban subsamples of Chinese schoolchildren. Overall, both subsamples have similar gender structures (49% females in urban areas and 50% females in rural areas), whereas urban children are younger (t-stat = −34.9, *p*-value < 0.001) than their rural peers. Notably, the youngest age group (10–11 years old) is underrepresented in the rural subsample dramatically (7%), while the oldest group (14–15 years old) comprises more than half of the subsample. Conversely, in the urban subsample, only 9% of children fall in the oldest age group. The sample difference in age structure reflects the corresponding population difference evoked by the recent decrease in the birth rate in rural Chinese areas. As would be expected, urban schoolchildren come from more educated families. 50% of rural mothers do not complete high school, while among urban mothers, the proportion of the lowest educational level is less than 25%.

**Table 1:** Socio-demographic description of rural and urban subsamples.

Variable		Urban areas	Rural areas
*N*		1,412	1,032
Gender	Male	718 (51%)	514 (50%)
	Female	694 (49%)	518 (50%)
Age	Mean (±SD)	12.04 (±1.07)	13.63 (±1.16)
	10–11 years old	449 (32%)	76 (7%)
	12–13 years old	839 (59%)	300 (29%)
	14–15 years old	124 (9%)	656 (64%)
Mother’s education	Below high school	331 (23%)	521 (50%)
	High school	259 (18%)	145 (14%)
	Secondary education	54 (4%)	19 (2%)
	Bachelor	92 (7%)	38 (4%)
	Master	152 (11%)	30 (3%)
	Doctor	19 (1%)	1 (0%)
	Other/Not clear	505 (36%)	278 (27%)

Total *N* = 2,444.

### 3.1 Math achievements, math anxiety, and nonverbal intelligence prevalence within age groups in rural and urban areas

**Figure 1:**
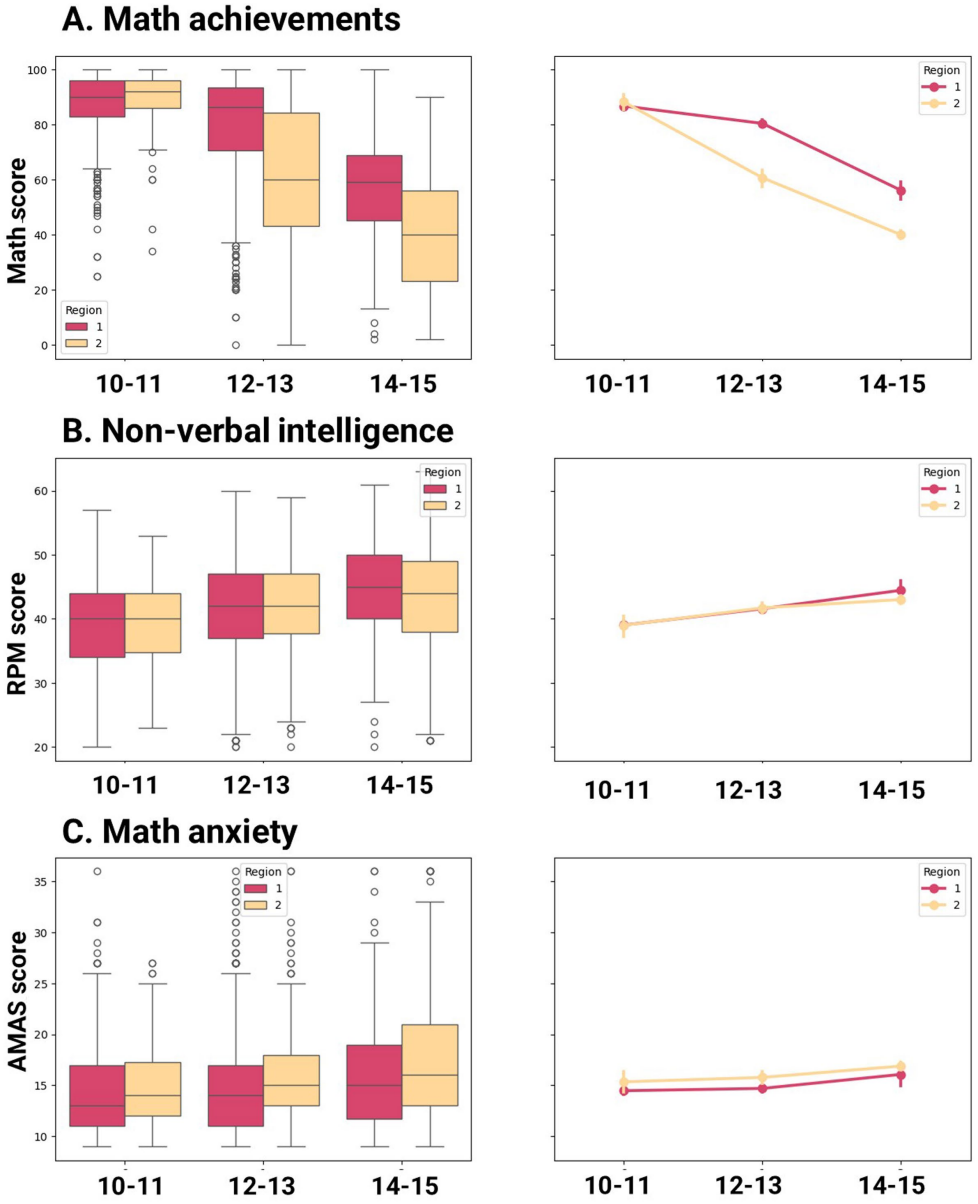
Differences in math achievements, RPM score and AMAS score between urban and rural area for different ages.

**Figure 2:**
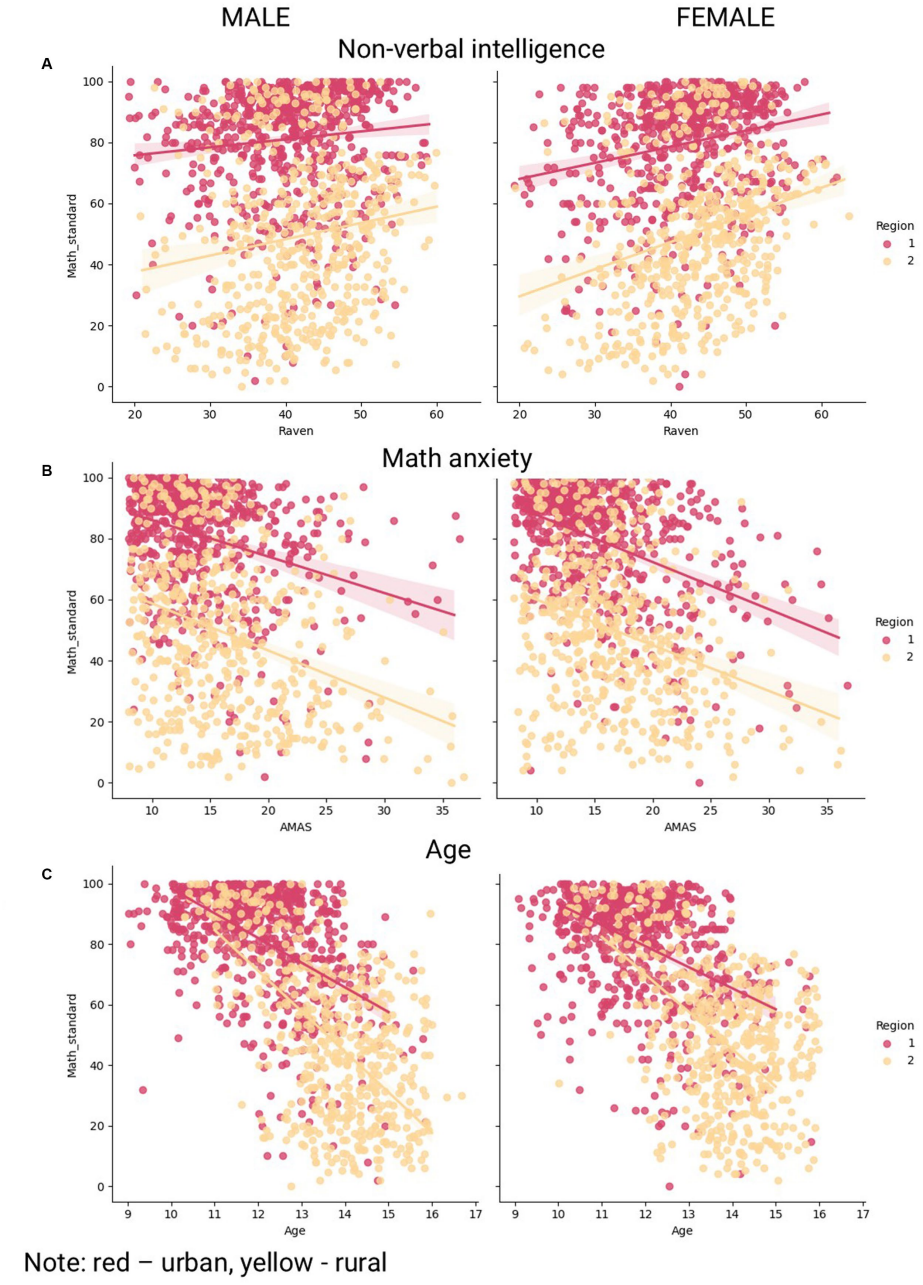
Non-verbal intelligence, math anxiety and age as correlates of math achievements.

Table 2 and Figure 1 show differences in math achievement, math anxiety, and nonverbal intelligence between urban and rural children across age groups. Regarding math achievement, no significant differences are observed in the youngest age group, while older urban children demonstrate an advantage over their rural counterparts. In other words, the achievement gap between rural and urban schoolchildren becomes more pronounced with age. Overall, the mean math score deteriorates with age for both subsamples, but for rural schoolchildren, the decline is more dramatic and starts earlier. Thus, the mean difference between the youngest and oldest age groups in urban areas is 30.6 points (t-stat = 21.55, *p*-value = <0.001), whereas in rural areas it is 48.24 points (t-stat = 20.99, *p*-value < 0.001). Interestingly, variance in math achievements increases substantially with age for both subsamples (see Figure 2). As for nonverbal intelligence, no differences are observed between rural and urban schoolchildren regardless of their age. For both groups, RPM score increases with age. Differences in AMAS between rural and urban schoolchildren are observed only for 12–13 years old (rural children show higher scores), but not for 10–11 years old and 13–14 years old. For both groups, math anxiety slightly increases with age.

**Table 2:** Differences in math achievements, RPM score, and AMAS score between urban and rural areas for different ages.

Variable	Urban mean (±SD)	Rural mean (±SD)	Difference (effect size)	T-stat	*p*-value
10–11 years old					
Math achievements	86.7 (±13.45)	88.3 (±11.31)	−1.4	−0.9	0.3
Raven	39.0 (±7.77)	39.0 (±6.94)	0.0	0.03	0.97
AMAS	14.5 (±4.57)	15.3 (±4.33)	−0.8	−1.5	0.1
12–13 years old					
Math achievements	80.4 (±17.53)	60.7 (±25.67)	19.7	14.7	<0.001**
Raven	41.5 (±7.54)	41.6 (±7.46)	−0.1	−0.25	0.8
AMAS	14.7 (±4.60)	15.8 (±4.86)	−1.1	−3.4	<0.001**
14–15 years old					
Math achievements	56.1 (±19.00)	40.0 (±19.59)	16.2	8.44	<0.001**
Raven	44.5 (±8.60)	43.0 (±8.00)	1.44	1.8	0.06
AMAS	16.1 (±5.66)	16.9 (±5.52)	−0.8	−1.5	0.1

Total *N* = 2,444. For within-group *N*, see Table 1. *p-value < 0.05, **p-value < 0.01.

### 3.2 Gender differences in rural and urban areas

Table 3 illustrates gender differences in math achievement, nonverbal intelligence, and math anxiety for urban and rural subsamples. Overall, there are no gender differences in math achievements in both cultural contexts. However, within the group of youngest urban schoolchildren (10–11 years old), boys slightly surpass girls in math. In both subsamples, girls outperform boys in nonverbal intelligence, with a higher effect size in urban areas. Meanwhile, in both urban and rural areas, girls demonstrate higher math anxiety levels, but in rural areas, this difference does not reach a threshold of statistical significance (*p*-value = 0.07).

**Table 3:** Gender differences in math achievements, RPM score, and AMAS score in rural and urban areas.

Variable	Male mean (±SD)	Female mean (±SD)	Difference (effect size)	T-stat	*p*-value
Urban areas (all ages)					
Math achievements	81.12 (±18.10)	79.49 (±18.02)	1.63	1.69	0.09
Raven	40.40 (±7.67)	41.67 (±7.55)	−1.27	−3.13	0.002**
AMAS	14.15 (±4.61)	15.36 (±5.01)	−1.21	−4.74	< 0.001**
Rural areas (all ages)					
Math achievements	49.26 (±26.59)	49.89 (±24.36)	−0.63	−0.40	0.69
Raven	41.83 (±7.72)	42.84 (±7.54)	−1.01	−2.11	0.03*
AMAS	16.15 (±5.52)	16.75 (±5.07)	−0.42	−1.80	0.07
Urban areas (10–11 years old)					
Math achievements	88.67 (±10.64)	85.02 (±13.45)	3.65	3.18	0.002**
Raven	38.71 (±7.46)	39.30 (±7.11)	−0.58	−0.85	0.39
AMAS	13.97 (±4.17)	14.93 (±4.82)	−0.96	−2.25	0.02*
Rural areas (10–11 years old)					
Math achievements	90.29 (±9.14)	85.45 (±15.37)	4.84	1.72	0.09
Raven	38.36 (±6.46)	39.84 (±7.09)	−1.48	−0.94	0.34
AMAS	14.68 (±4.27)	16.25 (±4.94)	−1.57	−1.47	0.14
Urban areas (12–13 years old)					
Math achievements	81.22 (±17.21)	79.61 (±17.85)	1.61	1.33	0.18
Raven	40.70 (±7.43)	42.51 (±7.35)	−1.81	−3.55	<0.001**
AMAS	14.19 (±4.55)	15.33 (±4.91)	−1.22	−3.74	<0.001**
Rural areas (12–13 years old)					
Math achievements	59.91 (±26.90)	61.39 (±24.61)	−1.49	−0.50	0.62
Raven	40.73 (±6.56)	42.56 (±7.65)	−1.83	−2.21	0.03*
AMAS	15.89 (±5.28)	15.69 (±3.93)	0.22	0.42	0.67
Urban areas (14–15 years old)					
Math achievements	81.22 (17.21)	79.61 (17.85)	1.61	1.32	0.18
Raven	40.70 (7.43)	42.51 (7.35)	−1.81	−3.54	<0.001*
AMAS	14.10 (4.55)	15.33 (4.91)	−1.22	−3.75	<0.001*
Rural areas (14–15 years old)					
Math achievements	59.91 (±26.90)	61.39 (±24.61)	−1.49	−0.50	0.62
Raven	40.73 (±6.56)	42.56 (±7.65)	−1.83	−2.21	0.03*
AMAS	15.89 (±5.28)	15.69 (±3.93)	0.22	0.42	0.67

Total *N* = 2,444. For within-group *N*, see Table 1. *p-value < 0.05, **p-value < 0.01.

### 3.3 The difference in the link between math anxiety, nonverbal intelligence, maternal education, and math achievements between urban and rural areas

Table 4 provides the results of linear regression models that were fit to urban and rural subsets separately. Adjusted R-squared, indicating the proportion of explained variance, is similar for both areas—0.244 and 0.293 for urban and rural schoolchildren, respectively. In the urban subpopulation model, the intercept is 63.45. It reflects an average math score for the baseline group of urban children—namely, those with average age, high math anxiety, low nonverbal intelligence, and low maternal education. The baseline group of rural schoolchildren scores 11 points lower (the model intercept is 52.44). High and medium nonverbal intelligence are positively associated with math achievement in both areas: for urban schoolchildren with medium and high intelligence scores, the differences from the baseline group are 8.31 and 17.24 points, respectively; for rural schoolchildren, those values are 10.71 and 18.97. Low and medium math anxiety are also positively linked with math in both contexts: the coefficient values are 16.05 and 10.42 for urban schoolchildren; 17.64 and 13.23 for rural schoolchildren, respectively. Maternal education was positively linked with math achievements for urban schoolchildren (urban schoolchildren with high maternal education score 6.88 points higher than the baseline group), but not for rural schoolchildren.

**Table 4:** Linear regression with interactions.

Variable	Urban		Rural	
	Coefficient [95% CI]	*p*-value	Coefficient [95% CI]	*p*-value
Age	−6.87 [−7.98; −5.76]	<0.001**	−10.15 [8.32; 11.98]	<0.001**
High nonverbal intelligence	17.24 [15.67;18.81]	<0.001**	18.97 [15.76; 22.18]	<0.001**
Medium non-intelligence	8.31 [6.91; 9.71]	<0.001**	10.71 [7.82; 13.60]	<0.001**
Low math anxiety	16.05 [14.61; 17.49]	<0.001**	17.64 [14.73; 20.55]	<0.001**
Medium math anxiety	10.42 [8.91; 11.93]	<0.001**	13.23 [10.34; 16.12]	<0.001**
High maternal education	6.88 [5.71; 8.05]	<0.001**	4.01 [0.79; 7.23]	0.23
Age* High nonverbal intelligence	4.59 [3.38; 5.80]	<0.001**	6.67 [4.43; 8.91]	<0.001**
Age* Medium non-intelligence	−0.14 [−1.11; 1.11]	0.87	3.01 [1.29; 4.73]	0.04*
Age* Low math anxiety	4.95 [3.87; 6.03]	<0.001**	−1.12 [−3.06; 0.82]	0.49
Age*Medium math anxiety	3.34 [2.15; 4.53]	<0.001**	−1.36 [−3.34; 0.62]	0.41
Age*High maternal education	2.05 [1.16; 2.94]	0.02*	0.93 [−1.41; 3.27]	0.69

Age was centered around the mean for that analysis. Baseline category: children with mean age, high math anxiety, low nonverbal intelligence, low maternal education. *p-value < 0.05, **p-value < 0.01. Total *N* = 1,661, as participants with unknown maternal education were removed from the analysis.

Additional correlation analysis reveals no differences in the magnitude of correlation between math anxiety and math achievement for rural and urban schoolchildren: r(urban) = −0.37**, r(rural) = −0.32**, z = 1.385, *p*-value = 0.17, as well as in the magnitude of correlation between nonverbal intelligence and math achievements: r(urban) = 0.16**, r(rural) = 0.21**, z = −1.263, *p*-value = 0.21. Correlations between maternal education and math achievements are at a subthreshold significance level for both urban (r = 0.09, *p*-value = 0.07) and rural (r = 0.10, *p*-value = 0.05) contexts, with a similar magnitude.

### 3.4 Nonverbal intelligence, math anxiety, and maternal education as moderators of the association between math achievements and age

Table 4 provides information on interactions between nonverbal intelligence/math anxiety/maternal education and age, separately for urban and rural schoolchildren. Overall, in both cultural contexts, children with higher nonverbal intelligence exhibit a more gradual decline in math achievement with age than children in the reference group, after adjusting for other variables. Thus, math achievement decline for urban schoolchildren in the baseline group is 6.87 points per year, but for their counterparts with high nonverbal intelligence, it is 2.28 points per year. For the rural subpopulation, the values are 10.15 and 3.48 points per year, respectively. Notably, an interaction “age * medium nonverbal intelligence” was significant for the rural subpopulation only with a near-threshold significance. An interaction between math anxiety and age was significant for urban, but not rural, schoolchildren. In the urban subpopulation, the groups of children with medium and low math anxiety levels (with other variables fixed) demonstrate a smoother math achievement decline (3.53 and 1.92 points per year, respectively) in comparison with a baseline group. An interaction between age and maternal education reached subthreshold significance for urban subpopulation (the achievement decline in urban schoolchildren with high maternal education is 2.05 points per year less in comparison with a baseline group) but was non-significant in urban context.

**Figure 3:**
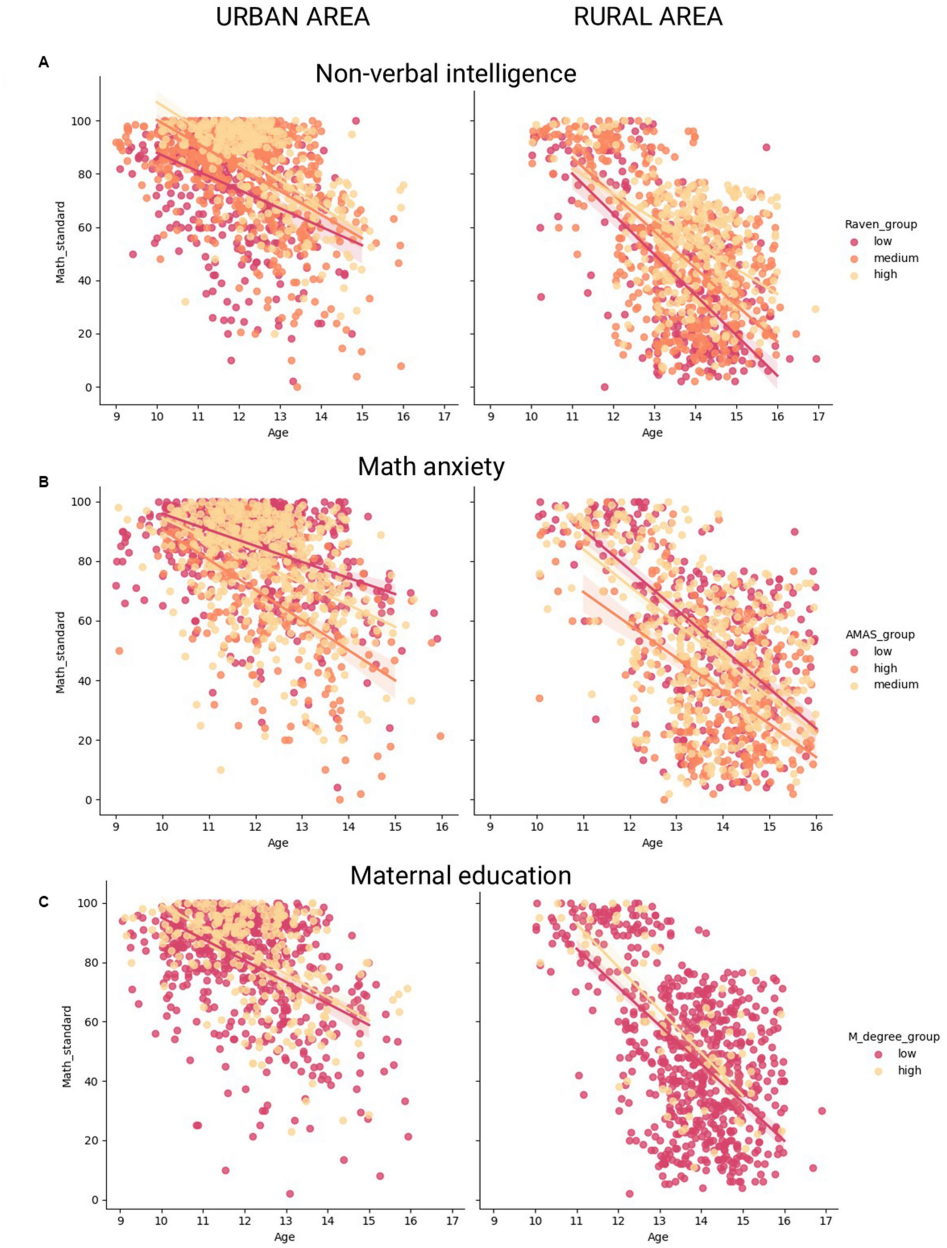
Association with math achievements and age for urban and rural subgroups with different non-verbal intelligence, math anxiety and mother’s education level.

Additional correlation analysis presented in Table 5 overall confirms the results obtained in the linear regression model with interactions between variables. Thus, there is a significant difference between the correlation coefficients obtained in the low- and high-nonverbal intelligence groups in both rural and urban areas (z = 4.611, *p*-value < 0.001, and z = 4.256, *p*-value < 0.001, respectively). No significant differences between correlation coefficients obtained within low, medium, and high math anxiety subgroups are observed for rural schoolchildren. For urban schoolchildren, however, there is a significant difference between correlation coefficients obtained within low- and high-math-anxiety subgroups (z = 2.79, *p*-value = 0.005). As for maternal education, there are no significant differences in correlation coefficients within low and high maternal education subgroups in both contexts. Figure 3 illustrates the results of the correlation analysis.

**Table 5:** Correlations between math achievements and age for urban and rural subgroups with different nonverbal intelligence, math anxiety, and mother’s education level.

Variable	Rural areas			Urban areas		
	Low	Medium	High	Low	Medium	High
Nonverbal intelligence						
r	−0.69	−0.64	−0.42	−0.62	−0.53	−0.38
95% CI	[−0.691; −0.689]	[−0.641; −0.639]	[−0.421; −0.419]	[−0.621; − 0.619]	[−0.531; − 0.528]	[−0.382; −0.378]
*p*-value	<0.001**	<0.001**	<0.001**	<0.001**	<0.001**	<0.001**
Math anxiety						
r	−0.53	−0.59	−0.62	−0.40	−0.42	−0.53
95% CI	[−0.531; −0.529]	[−0.591; −0.589]	[−0.621; −0.619]	[−0.401; −0.399]	[−0.421; −0.419]	[−0.531; −0.529]
*p*-value	<0.001**	<0.001**	<0.001**	<0.001**	<0.001**	< 0.001**
Mother’s education						
r	−0.59	–	−0.59	−0.45	–	−0.44
95% CI	[−0.591; −0.589]	–	[−0.591; −0.589]	[−0.451; −0.449]	–	[−0.441; −0.439]
*p*-value	<0.001**	–	<0.001**	<0.001**	–	<0.001**

*p-value < 0.05, **p-value < 0.01. Total *N* = 1,661, as participants with unknown maternal education were removed from the analysis.

## 4 Discussion

The purpose of the study was to investigate the interplay among socio-demographic variables, math anxiety, and nonverbal intelligence, and to examine their association with math achievement among schoolchildren in urban and rural areas of China. Overall, despite no differences in nonverbal intelligence and subtle differences in math anxiety between rural and urban areas, urban schoolchildren dramatically outperform their rural counterparts in math, with the highest effect size of almost 1 standard deviation for the oldest age group. In previous studies, the math achievement gap between urban and rural areas was also observed for Jordanian (Ababneh and Kodippili, 2020), Peruvian, Indian, Vietnamese (Betancur et al., 2023), Pakistani (Butt and Dogar, 2014; Tayyaba, 2012), and Chinese (Wang et al., 2020) schoolchildren. A review (Williams, 2005) shows that the urban–rural math achievement gap is observed across 14 countries out of 24. Possible explanations include lower family SES (Williams, 2005), lower academic commitment and career aspirations (Reeves, 2012), the lack of access to early childhood education (Betancur et al., 2023) and to advanced math courses (Irvin et al., 2017), the lack of federal funding and high-quality teachers in rural areas (Graham and Provost, 2012), as well as better school environment in big cities, such as higher emphasis on achievements and discipline (Ababneh and Kodippili, 2020). Moreover, lower quality of rural schools may lead to a negative perception of the school environment by rural schoolchildren, resulting in school refusal and underachievement (Sorrenti et al., 2024). Lack of instructional quality in rural schools may also hinder achievement motivation in math (Scherer and Nilsen, 2016).

In both cultural contexts, nonverbal intelligence shows a moderate positive association with math achievement, with no substantial difference in magnitude between rural and urban schoolchildren (r = 0.21 and r = 0.16, respectively). These results are consistent with previous findings on the association between RPM score and math achievement in schoolchildren in Eastern Morocco (r = 0.381; Bouchefra et al., 2022) and with a meta-analysis showing r = 0.41 between fluid intelligence and math achievement across different studies (Peng et al., 2019). On the other hand, math anxiety shows a moderate negative association with math achievement, with no differences for rural and urban areas (r = −0.32 and r = −0.37, respectively). Similar results were obtained in previous studies. Thus, two recent meta-analyses (Ulum and Küçükdanaci, 2022; Barroso et al., 2021) provide the estimates of r = −0.42 and r = −0.28, respectively. A negative link between math anxiety and math performance was also observed in the Chinese cultural context (Fu, 2025). Maternal education was shown to be positively yet weakly associated with math achievements in urban, but not rural, schoolchildren. The absence of the maternal education effect may be partially explained by the underrepresentation of parents with higher education in rural areas or inaccurate measurement. Furthermore, one previous study reports a stronger link between family SES and math achievements for urban schoolchildren (Miller et al., 2013). Presumably, in a rural context, even educated parents do not have enough time, resources, or aspiration to provide their children with a stimulating learning environment at home, leading to their inability to improve the math achievements of their offspring.

In rural areas, math achievement deteriorates dramatically with age, whereas in urban China, this deterioration is less pronounced, leading to a widening of the achievement gap over time. The continuous enlargement of the rural/urban gap was observed in a previous study on US schoolchildren (Graham and Provost, 2012). The low quality of rural schools and their lack of monetary and human resources may contribute to such a dynamic (Graham and Provost, 2012), as well as lower achievement aspirations in the rural population (Reeves, 2012).

Notably, high nonverbal intelligence and low or medium math anxiety are negatively associated with deterioration of math achievement with age in urban populations. Thus, there is a weaker association between achievement and age in subgroups of urban schoolchildren with high nonverbal intelligence or low or medium math anxiety, whereas a stronger negative association is observed for the rest. Low math anxiety is positively associated with higher math-specific grit (i.e., readiness to put efforts into math learning) (Yu et al., 2021) and enjoyment of mathematics (Dowker et al., 2016). On the other hand, high math anxiety leads to avoidance of math (Ashcraft, 2002), procrastination in performing math work (Yu et al., 2021), and an “affective drop” (Ashcraft and Moore, 2009). Presumably, children with low math anxiety are more likely to maintain aspiration for high math achievements, which hinders the decline of a math score with age. Conversely, high nonverbal intelligence may help to manage more complex math tasks that emerge in higher grades (Miller Singley and Bunge, 2014).

Interestingly, for the rural subpopulation, only high nonverbal intelligence attenuates the negative link between age and achievement, while math anxiety level does not moderate the relationship. One possible reason for the lack of the math anxiety effect in rural areas is a much stronger impact of other negative factors, such as poor quality of teachers and low learning motivation. While interventions aimed at reducing math anxiety may be effective in preventing math achievement decline in cities, they may not be effective in rural areas due to specific obstacles. Fostering the quality of the teaching practices and positive school environment may be a more efficient intervention for rural schools (Scherer and Nilsen, 2016; Sorrenti et al., 2024).

Meanwhile, age shows a positive association with fluid intelligence for both subgroups, reflecting the development of working memory and abstract reasoning skills (Chan, 2004). Math anxiety slightly increases with age as well, as was shown in our previous study (Linna et al., 2024).

Regarding gender differences, in the youngest age group, boys surpass girls in math in urban areas, which is consistent with several studies on the gender gap (Ellison and Swanson, 2010; Else-Quest et al., 2010; Cimpian et al., 2016; Reilly et al., 2015), while no gender differences are observed for other groups. On the contrary, in both rural and urban areas, girls outperform boys in nonverbal intelligence. Previous studies have demonstrated that, at a younger age, girls surpass boys in working memory (Niazi and Adil, 2021) and fluid intelligence (Buczyłowska et al., 2019) tasks, but the difference becomes negligible with age (Buczyłowska et al., 2019). At the same time, in urban areas, girls exhibit higher math anxiety, whereas no gender differences in math anxiety level are observed for the rural subsample. Higher prevalence of math anxiety in girls was demonstrated by numerous studies (Marakshina et al., 2023, 2024; Xie et al., 2019; Schmitz et al., 2022; Primi et al., 2014; Sadiković et al., 2018; Linna et al., 2024). Further investigations are needed to explore why gender differences in math achievements and math anxiety are not pronounced in rural Chinese schoolchildren. One possible reason is that, in rural areas in general, there is less cultural emphasis on math achievement, which makes gender stereotypes about math less salient.

Several limitations have to be acknowledged. First of all, a cross-sectional design does not allow us to observe developmental trajectories directly and purely distinguish the developmental effect from the cohort effect. Further longitudinal studies on the interplay among math achievement, math anxiety, nonverbal intelligence, and socio-demographic variables are needed to justify our findings. Another possible direction for future studies is to investigate the origins of the rural/urban achievement gap in the Chinese context, the reasons for its substantial growth with time, and the effectiveness of different interventional strategies aimed at reducing the gap. Longitudinal and interventional studies may be particularly useful for this purpose.

Age imbalance between rural and urban areas should also be considered a limitation, although age was controlled in the analysis. The number of young students in rural areas is small, mainly because of the impact of the birth rate.

Overall, the current study replicates several well-established findings (i.e., a positive association between math achievement and nonverbal intelligence, a negative association between math achievement and math anxiety, a math achievement gap between rural and urban areas, and higher math anxiety and nonverbal intelligence in girls) for the cultural contexts of rural and urban China. Meanwhile, the study also reveals some intricate differences between rural and urban areas that bring novelty to the field, such as the presumably protective effect of low math anxiety on math achievement decline for urban but not rural schoolchildren and more pronounced gender differences in urban areas in comparison with rural regions.

## Data Availability

The raw data supporting the conclusions of this article will be made available by the authors, without undue reservation.
